# Pupal diapause termination in *Bactrocera minax*: an insight on 20-hydroxyecdysone induced phenotypic and genotypic expressions

**DOI:** 10.1038/srep27440

**Published:** 2016-06-08

**Authors:** Zhenzhong Chen, Yongcheng Dong, Yaohui Wang, Awawing A. Andongma, Muhammad A. Rashid, Patcharin Krutmuang, Changying Niu

**Affiliations:** 1 College of Plant Science & Technology, Huazhong Agricultural University, Wuhan 430070, China; 2 Department of Entomology & Plant Pathology, Faculty of Agriculture, ChiangMai University, ChiangMai 50200, Thailand

## Abstract

The Chinese citrus fruit fly, *Bactrocera minax*, is an economically important pest of citrus. It exhibits pupal diapause from November to May to combat harsh environmental conditions. Such a long pupal diapause is a barrier for laboratory rearing and development of control strategies against this pest. In the present study, 20-hydroxyecdysone (20E) was used to break pupal diapause of *B. minax* by topical application. After diapause termination by 20E treated, the pupal ontogenetic processes were observed along the temporal trajectory. The pupal response time to 20E was estimated by detecting the relative expression of 20E responsive genes at different times after 20E-treatment. Results revealed that 20E could effectively terminate the pupal diapause in a dose-dependent manner and significantly shorten the time for 50% adult emergence (Et_50_). 20E response genes, including *ecr*, *broad* and *foxo*, were up-regulated within 72h, indicating these genes are involved in pupal metamorphosis and diapause termination processes. Morphological changes showed the pupal metamorphosis began ~7 days after 20E-treatment at 22 °C. This study does not only pave the way for artificial rearing in the laboratory through manipulating of pupal diapause termination, but also deepens our understanding of the underlying pupal diapause termination mechanism of *B. minax*.

A salient feature of insect’s life cycle is diapause, which is a genetically determined response to environmental stimuli, such as dehydration, extremely low or high temperatures, scarcities of food and other hostile conditions^1^. Diapause is a dynamic developmental arrest, accompanied with metabolic depression and enhanced stress tolerance^1^,^2^. It also increases their chance of mating by synchronizing the growth rates of population which ultimately plays a vital role in insect survival^2^,^3^. There are two kinds of diapause (obligatory diapause and facultative diapause) and three type of eco-physiological phases including pre-diapause, diapause and post-diapause^4^,^5^. Presently, studies on pupal diapause such as in *Heliothis armigera*^6^, *Antheraea pernyi*^7^, *Delia antiqua*^8^, *Sarcophaga crassipalpis*^9^ and *Sarcophaga bullata*^10^, are getting more focus because it not only provides insights into physiological mechanisms regulating diapause but also potentially offers methods for manipulating laboratory colonies to facilitate rearing or for disrupting field populations in pest management programmes^11^.

In insects, hormones like juvenile hormone (JH) and molting hormone (MH) play a role in regulating their metamorphosis, reproduction and development including diapause and these hormones are inhibitive to each other in insect metamorphosis^12^,^13^. Pupal diapause is characterized by a shutdown of the brain-prothoracic gland (PG) axis as it fails to synthesis and release the ecdysteroids needed to promote development^1^. Pupal diapause can be terminated by different ways, including environmental cues, chemicals and hormonal stimuli. Although the environmental stimulation method is the most popular^1^,^4^,^5^,^9^,^14^, hormones such as 20E, JH and diapause hormone (DH) usually provided other tricks to manipulate diapause. They break diapause in some insect species^15^,^16^,^17^,^18^,^19^, which indicates hormonal levels *in vivo* in insect directly determines the status of diapause, but the defining hormone is not usually constant in different species or developmental stages of insects. JH usually plays a role on egg and adult diapause^16^,^17^, whereas 20E affects egg, larval and pupal diapause^16^,^18^ and DH also controls pupal diapause^19^.

*Bactrocera minax* (Enderlein) (Diptera: Tephritidae) is an important economic pest of citrus in China and its neighboring countries^20^,^21^. It is univoltine with long pupal diapause, which is a great obstacle for artificial rearing and development of control strategies against this pest. Pupal diapause in *B. minax* is obligatory and cannot be terminated if directly exposed to high temperature or if organic chemicals are topically applied^22^,^23^. So far, several aspects of pupal diapause in the Chinese citrus fruit fly have been studied. Dong *et al.* investigated the effects of low temperature chilling on pupal diapause development and termination^23^ and transcriptome characterization analysis of different pupal stages^22^. Lü *et al.* characterized the diapause relative heat shock protein (Hsp) genes *hsp23*, *hsp70* and *hsp90*^24^. However, a developmental process triggered by 20E and the related early-response genetic expression associated with diapause termination remains largely unknown. In this study, different concentrations of 20E were used to terminate pupal diapause of *B. minax* by topical application. The pupal developmental process after diapause termination was observed by photomicrograph. Ecdysone nuclear receptor (EcR) and 20E response genes were also studied, including several transcription factors, such as hormone receptor 3 (*HR3*), earlier response genes (*E74A*) and Broad-Complex (*Br/Br-c*)^13^,^25^,^26^,^27^. The transcription factor *Br/Br-C* is reported to be a “pupal specifier” that initiates insect metamorphosis in *Manduca sexta* and *Drosophila melanogaster*^28^,^29^,^30^. Br-mediated hormone directly regulates stem cells to generate adult cells during metamorphosis^31^. In addition, previous studies reported the transcription factor Forkhead-box O (FOXO), a downstream molecule in the insulin and juvenile hormone signaling, normally expressed in the absence of insulin and well known to mediate the diapause in *Culex pipiens*^17^,^32^. The relative mRNA expression of these genes, including *ecr*, *foxo*, *broad*, *bhr3*, *e74a* and *vrille*^33^,^34^, were detected with quantitative real-time polymerase chain reaction (qRT-PCR) after pupae topically treated with 20E to further understand the mechanism of pupal diapause in *B. minax.*

## Results

### Breaking pupal diapause of *B. minax* by using 20E

**Figure 1:**
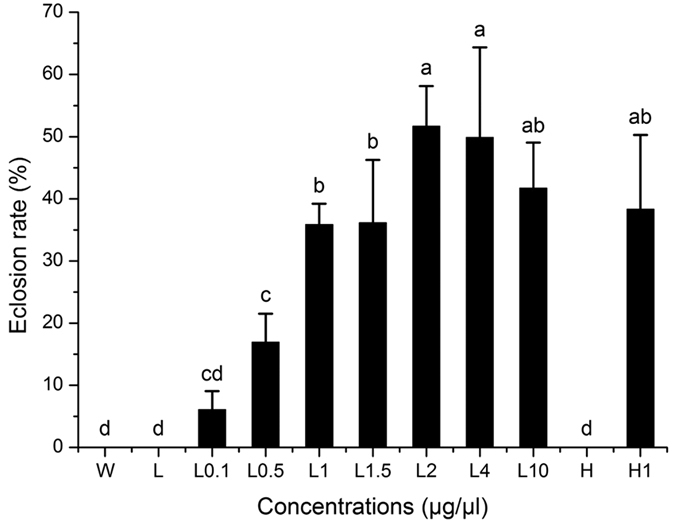
Means ± SD of *Bactrocera minax* pupal eclosion rate after treatment with control (W = water, L= 10% ethanol and H = 70% ethanol) and different concentration 20E (L0.1, L0.5, L1, L1.5, L2, L4 and L10 respectively equals to 0.1 μg/μl, 0.5 μg/μl, 1 μg/μl, 1.5 μg/μl, 2 μg/μl, 4 μg/μl and 10 μg/μl in 10% ethanol; H1 = 1 μg/μl in 70% ethanol) in November. Different letter shows significant difference (*p* < 0.05, ANOVA).

*B. minax* pupal diapause could be effectively terminated by topical application of 20E in a dose-dependent manner (Fig. 1). The eclosion rate was significantly raised with increasing concentrations of 20E in the range of 0.1 to 2 μg in 10% ethanol (Fig. 1). However, once the applied 20E concentration increased to more than 2 μg/μl, the eclosion rate tended to decrease. In addition using a 20E concentration of 1 μg/μl in 10% and 70% ethanol as a dilution solvent (L1 and H1 respectively) had no significant difference in their effects (Fig. 1). Similarly, the effects of the three controls including pure water (W), 10% ethanol (L) and 70% ethanol (H) were not significantly different when applied topically.

**Figure 2:**
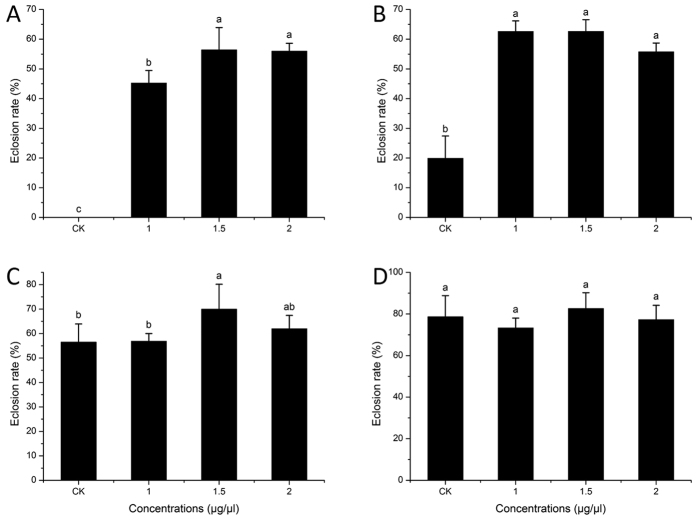
Means ± SD of *Bactrocera minax* eclosion rates after 20E-treatment in December (**A**), January (**B**), February (**C**) and March (**D**). Different letter shows significant difference (*p *< 0.05, ANOVA).The different concentrations were chosen after a preliminary study in November. These include 1 μg/μl, 1.5 μg/μl, 2μg/μl of 20E diluted in 10% ethanol and CK (10% ethanol).

Based on the above experiments carried out in November 2014, three best concentrations of 20E were chosen to break diapause in the next set of experiments in the following months of December, January, February and March (Fig. 2). Adult emergence from the different 20E treatments recorded in December (Fig. 2A) was similar to the results obtained in November. The highest eclosion rate was recorded at a concentration of 1.5 μg/μl. It was not significantly different from the 2 μg/μl concentration however both rates were significantly higher than the 1 μg/μl concentration. In January there was no significant difference between the 20E treatments (Fig. 2B), but the eclosion rate of the control (CK) unexpectedly increased. However, this increase was still significantly lower than the 20E treatments, indicating that a small part of wild pupae may gradually terminate diapause after transferred to 22 °C. The eclosion rates in the control continuously increased through the months of February and March. In February, the eclosion rate of the control was not significantly different from the 1 μg/μl and 2 μg/μl of 20E treatments (Fig. 2C), while in March the eclosion rates of all the treatments including the control were statistically the same (Fig. 2D), indicating the termination of pupal diapause.

In April it was realized that adult metamorphosis has started in some 20E-nontreated pupae, which were transferred into an incubator maintained at 22 °C. Their eclosion rate had no significant difference with the control in March.

### Improving synchronization of emergence using 20E

**Figure 3:**
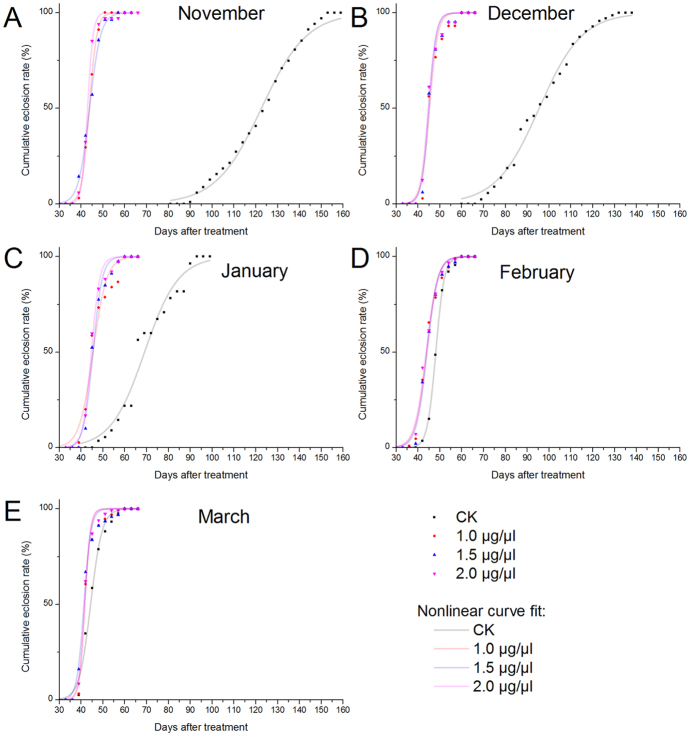
Cumulative eclosion rate of *Bactrocera minax* in November (**A**), December (**B**), January (**C**), February (**D**) and March (**E**) after treated with different concentrations of 20E (1, 1.5 and 2 μg/μl) and CK (10% ethanol). Pupae were collected from the wild every month from November 2014 to March 2015 and incubated at 22 °C after treatment. The adult emergence was recorded every day, but for ease of presentation data were presented as cumulative 3-day counts.

20E can break the pupal diapause of *B. minax* by significantly reducing pupal diapause developmental duration and synchronizing adult emergence (Fig. 3). The time for 50% adult emergence (Et_50_) in 20E treated groups were significantly shortened than controls in November, December and January (Fig. 3), about 80, 50 and 20 days respectively (Table 1). Three concentrations of 20E treated groups (1, 1.5 and 2 μg/μl) in every month were almost overlapped (Fig. 3), that is, in a similar adult eclosion pattern even though they caused statistical significance in eclosion rate in some groups e.g., November (Fig. 1) and December (Fig. 2A). Et_50_ in February and March were still statistically different between control and 20E treated groups (Table 1), although the sigmoidal curves were very close (Fig. 3). Both Et_50_ and starting day of emergence (SDE) in control groups were significantly reduced over the months because of the decreasing diapause intensity^5^,^23^, on the contrary they were kept constant about 45 and 40 days respectively in 20E treated groups (Table 1). Furthermore, the control SDE also had a significant difference between 20E-treated groups in November, December, January and February but not in March (Table 1).

### Pupal development induced by 20E

**Figure 4:**
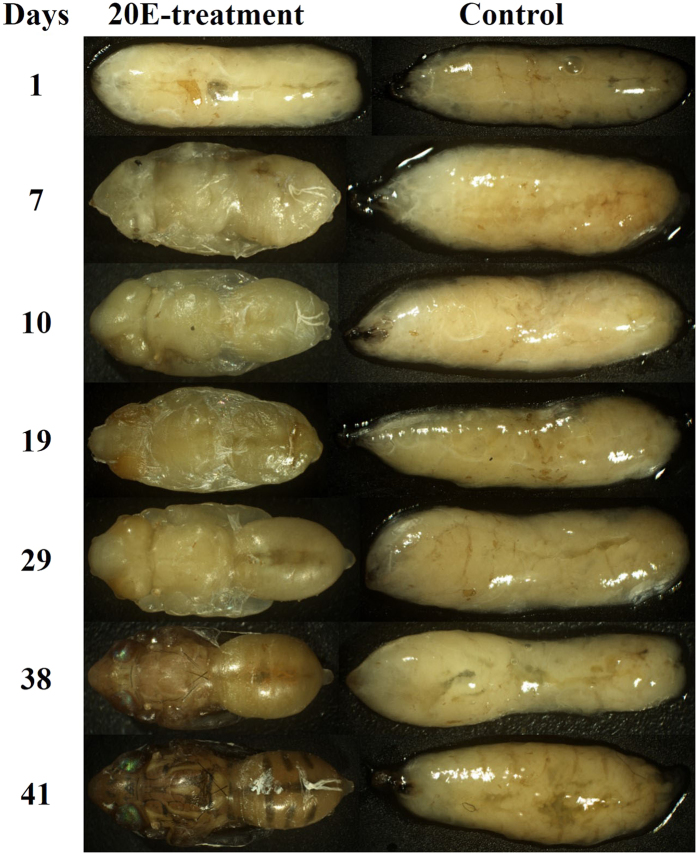
Photomicrographs of *Bactrocera minax* pupal ontogenetic processes after 20-hydroxyecdysone (20E) and control treatments. In each photomicrograph pupal development were shown after removing the puparium, includinig 1, 7, 10, 19, 29, 38 and 41 days, after treatment, respectively.

From preliminary data it was estimated that adult emergence commenced at ~40 days after 20E treated pupae were incubated at 22 °C. However there was still a gap regarding when metamorphosis exactly began in 20E treated pupae and their developmental process. Thus, photomicrographs were taken to record the developmental processes of pupae with and without 20E treatment. Despite the fact that the development of pupae was not synchronized, the earliest change occurred 5 days after 20E-treatment when most of pupae still had a larval morphology inside the puparium. Metamorphosis began in most 20E treated pupae 7 days after treatment (Fig. 4). Finally, the adults in the 20E treated groups emerged while the pupae of the control group still had a larval morphology inside puparium (Fig. 4).

On the tenth day after 20E treatment, the head, thorax and abdomen of insect, could clearly be distinguished and the color of body and eyes were milky (Fig. 4). On the twentieth day, the color of eyes gradually changed to light yellow and the body was wrapped by a layer of connective tissues (Fig. 4). On the thirtieth day, the legs and wings of the insect were formed, and the abdomen turned round and connective tissues disappeared (Fig. 4). Thereafter, on the fortieth day, the color of body switched to brown from head to abdomen and specific stripes of the *B. minax* adult appeared. The wings got darker and the eyes had metallic luster at the same time (Fig. 4). Finally, the legs of insect thoroughly separated from body and moved out of the body after which a little hole was opened on the anterior end of the pupa through which the adult emerged and spread its wings to fly (Fig. 4).

### The relative expression of 20E response genes

**Figure 5:**
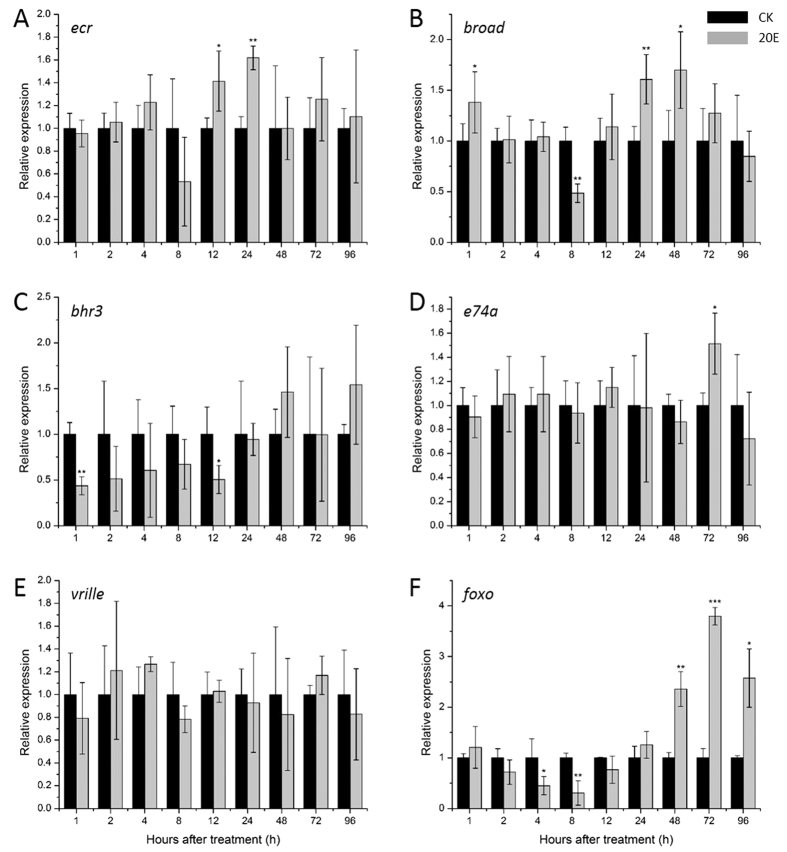
Relative mRNA expression of *ecr* (**A**), *broad* (**B**), *bhr3* (**C**), *e74a* (**D**), *vrille* (**E**) and *foxo* (**F**) after 20E and control (CK) treatments of *Bactrocera minax* pupae. Samples collected in 1 h, 2 h, 4 h, 8 h, 12 h, 24 h, 48 h, 72 h and 96 h, after 20E-treatment. Four replications were made. Asterisk shows significant difference (*p < 0.05, **p < 0.01 and ***p < 0.001, t-test).

The relative expression of *ecr*, the nuclear receptor of ecdysone, significantly upregulated at 12 h and 24 h but downregulated at 8 h after 20E-treatment (Fig. 5A). Surprisingly, the relative expression pattern of *broad* was very similar to *ecr*. They were significantly upregulated at 1 h, 24 h and 48 h and downregulated significantly at 8 h after 20E-treatment (Fig. 5B). Contrarily, the relative expression of a hormone receptor of *B. minax bhr3*, significantly decreased at 1 h and 12 h after 20E-treatment and then increased in 48 h and 96 h but this increase was not significant (Fig. 5C). On the other hand, *e74a*, an early response gene of 20E, had a significant increased expression at 72 h after 20E-treatment (Fig. 5D). Unexpectedly, the 20E response gene *vrille* had no significant difference of its relative expression after 20E-treatment (Fig. 5E). In addition to these genes, the relative expression of *foxo* also had a significant upregulation at 48 h, 72 h and 96 h and downregulation at 4 h and 8 h after 20E-treatment (Fig. 5F).

## Discussion

Insect’s secretion of prothoracicotropic hormone (PTTH) through brain stimulates PG to synthesize and release 20E which accelerates molting and metamorphosis^4^,^19^. The results from this study indicate that topical application of 20E on the *B. minax* pupae plays a crucial role in pupal diapause development and termination. An increase in 20E concentration from 0.1 to 2 μg led to a corresponding increase in eclosion rates (Fig. 1). In November when insects just went into diapause, the optimal 20E concentration which could be used to terminate diapause was 2 μg/μl (Fig. 1). However this optimal concentration dropped to 1.5 μg/μl in the latter months (Fig. 2). This suggests that, the pupae can synthesize 20E by themselves and once the titer of 20E *in vivo* arrives to a liminal value they will metamorphosize into the adults. Wang *et al.* studied the titer of endogenous 20E in different pupal stages and reported that, the 20E titer increased constantly throughout the pupal life stage^35^. During this stage, the optimal exogenous 20E required to terminate diapause probably decreased as a result of an increase in 20E concentration *in vivo*. In the early pupal stage, the concentration of 20E *in vivo* is low resulting in a decreased metabolism hence the pupae remains in diapause throughout the unfavorable winter condition. Exogenous 20E topically administered to the pupae, increased the 20E concentration and activated the 20E responsive genes, and finally triggered diapause termination. Nevertheless, when the 20E concentration increased beyond the threshold value, the eclosion rates did not increase (Fig. 1).

**Figure 6:**
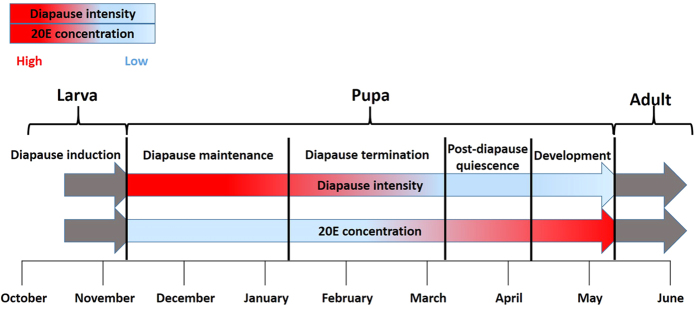
Schematic illustration of *Bactrocera minax* pupal diapause intensity and 20E concentration during different pupal ontogenetic processes. The first line indicates diapause intensity and second line shows 20E concentration. The red colour means the high diapause intensity and the high concentration of 20E, whereas the light blue colour is on the contrary.

It has been reported that low chilling temperature has a significant effect on pupal diapause development and termination^23^. Low temperature has also been reported to moderate the 20E contents in some herbal plant, whereby a decrease in temperature led to an increase in 20E levels in the plant and *vice versa*^36^. In this experiment, non-treated pupae collected in November and December and which were exposed to little or no winter chilling could not emerge in the laboratory after they were incubated at higher temperatures of 22 °C. Contrarily, wild pupae collected in January (which had been exposed to winter chilling for about 2 months) emerged after incubation. This suggests that, the 20E concentration *in vivo* of the pupae collected in January, had arrived the threshold value and therefore they could successfully terminate diapause, while those collected in November and December, were still at a diapause maintenance stage (Fig. 6). In other words, the return of suitable environmental conditions after a chilling phase stimulates diapause termination^23^. From these results it is tempting to believe that low chilling temperature might be the driving force that leads to the production of 20E *in vivo*. However further research needs to be carried out to confirm this hypothesis.

There were no significant difference in the pupal eclosion rates in the warmer months of March and April, which indicates that most of the pupae might have finished diapause. Therefore pupal diapause termination in *B. minax* occurs from January to March (Fig. 6). Even though wild pupae can successfully terminate diapause during this time, low environmental temperatures inhibit continuous development of the pupae. Thus they entered into post-diapause quiescence stage (Fig. 6). At this stage, the pupae can develop into adults once the environmental conditions are suitable. Therefore, the effects of 20E on pupal diapause termination is the most obvious in early periods of diapause (November and December) when diapause intensity at its maximum^5^, whereas moderately become obscure in later periods since the diapause intensity is gradually declining^5^,^23^ and this period is close to post-diapause quiescence stage. Finally in April, the environmental temperatures become warmer and suitable for metamorphosis to occur (Fig. 6).

Suitable environmental temperatures in combination with an optimal 20E concentration *in vivo*, causes the pupal diapause intensity to decrease gradually^22^. Although pupal diapause intensity is gradually decreased with the pupal development^22^, our results indicate that the diapause intensity could be different in different individuals. As a result, only those pupae that have lower diapause intensities could successfully emerge in January in the control group. However this emergence was significantly lower when compared with the groups treated with 20E (Fig. 2B). In the warmer months of February and March, there was no significant difference in the emergence between the control and 20E treated groups. This suggests that most of the pupae have high diapause intensity which lead to long diapause duration. This long diapause duration provides a convenience to *B. minax* as an invasive insect pest once adults are exposed to suitable environmental conditions and abundance of food. In addition long diapause in most individuals assist in synchronizing the population so it is easy to find mating partners.

From our results, exogenous 20E can not only break pupal diapause of *B. minax* but also improve synchronism of adult emergence for diapause-terminated pupae (Table 1). The Et_50_ of the CK group in March was significantly longer than 20E-treated groups and there was no significant difference in SDE for these groups, which indicate both of 20E and low temperature chilling can make a contribution to the synchronism of adult emergence^23^. Synchronization of adult emergence with the help of 20E provides convenience since synchronization of adult emergence could lead to mass production of *B. minax* adults in the future which could be used for research and pest management programs such as the sterile insect technique (SIT). In addition this knowledge could act as a foundation for artificial rearing of *B. minax* in the laboratory which till date is still impossible.

Based on our results, pupal metamorphosis starts about one week after topical application of 20E and this process could be broken down into 5 different steps. 1) Mouth hook comes out from puparium and initial shaping into adult morphology; 2) The formation of legs, wings and colour change of compound eyes; 3) Pigment deposition including body coloration from white to puce or dark, and eyes turned metallic luster; 4) Appearance of adult-like features and separation of the legs from the body; 5) Stretching of wings to fly.

20E is an important decisive factor which regulates genes associated with diapause termination. As shown in Fig. 5, relative expressions of some 20E responsive genes, such as *ecr*, *broad*, *e74a* and *foxo*, up-regulated after 12 h when 20E was topically applied. However, before 12 h the relative expression of some genes, such as *broad*, *bhr3* and *foxo*, unexpectedly down-regulated in some points. It is not clear about the mechanism involved. These downregulations may result from either stress/immune response triggered by 20E or resistance to exogenous 20E. As reported in Beckstead *et al.*, when the 20E concentration increased, the relative expression of *ecr* decreased firstly and increased subsequently^33^. Similar case in RNAi experiment, the relative expressions of target genes were expected to be down-regulated, however, some previous studies report that some genes up-regulated first and significantly down-regulated subsequently after RNAi which might refer to insect immunity towards exogenous stimulus^37^. Based on qRT-PCR results, there are possible ways through which 20E regulates diapause. That is, 20E could bind to its receptor-complex EcR/Usp and induce FOXO mRNA expression thereby increasing its transcriptional activity. Overexpression of FOXO upregulates two lipase genes — the insect adipose triacylglycerol lipase homologue *brummer* and an acid lipase, *acid lipase-1*^38^. Sequentially, these two genes promoted lipolysis in the fat body. They reduce the lipid content of the fat body and double the lipid concentration in hemolymph^38^. This in turn, accelerated pupal development and diapause termination. On the other hand, 20E could upregulate *broad*, a major “pupal specifier” in *Drosophila* and *Tribolium*^29^,^39^. Konopova *et al.* demonstrated that *broad* is an ecdysteroid-response gene and it is necessary for the differentiation of pupal characters by using RNAi in *Tribolium*^40^. Zeng *et al.* reported that *broad* relays hormone signals and regulates stem cells to generate adult cells during metamorphosis in *Drosophila* midgut^31^.

In conclusion, our studies suggested that 20E topical application could terminate pupal diapause of *B. minax* in a short time and improve the adult synchronization. These works are useful to better understand the mechanism of pupal diapause in *B. minax*. Such an understanding could allow us to develop new approaches of management against this insect pest.

## Materials and Methods

*Bactrocera minax* used in this study was collected from citrus orchards in San Douping (Latitude: 30.821, Longitude: 111.051), Hubei province, China. Pupae collected every month from December 2014 to April 2015, whereas larvae collected only during November 2014. 20E (C_27_H_44_O_7_) was purchased from Aladdin^35^ and ethanol used as the dilution solvent was obtained from Sinopharm Chemical Reagent Co., Ltd (China).

### Termination of pupal diapause of *B. minax* by using 20E

To break the pupal diapause in *B. minax,* different concentrations of 20E were used ranging from 0 μg/μl to 10 μg/μl in 10% and 70% ethanol. These were applied topically on the puape. Ethanol was chosen as a dilution solvent because 20E is insoluble in water. In this set of experiments each treatment was applied to a set of 30 insects and replicated 5 times (N = 150). A 20E stock solution of 100 μg/μl was first prepared which was later diluted to the required working concentrations of 10 μg/μl, 4 μg/μl, 2 μg/μl, 1.5 μg/μl, 1 μg/μl, 0.5 μg/μl and 0.1 μg/μl in 10% ethanol solvent and 1 μg/μl of 70% ethanol solvent. The control treatments included pure water, 10% and 70% ethanol.

Initially, pupae were obtained by placing mature third instar larvae of *B. minax* collected from wild into sterile sand to pupate in mid-November 2014. The pupae obtained were transferred to sand in a diameter = 90 mm petri dish. Then 1 μl of the different concentrations of 20E was applied on each pupa using a micropipette. The petri dishes containing the pupae were later placed into an incubator at 22 ± 1 °C with a relative humidity (RH) of 70 ± 5%^23^. Finally, distilled water was moderately sprayed when deemed necessary to maintain the humidity (70 ± 5%) until the pupae emerged after approximately 40 days. For measuring the effect of terminating diapause, the eclosion rate was calculated within two months after treatment.

Similarly, *B. minax* pupae, which were collected from wild in December 2014, January, February and March of 2015, were also topically treated with different concentrations of 20E and incubated under the same condition (22 ± 1 °C, RH =  70 ± 5%). However in this case, only the three optimal concentrations of 1 μg/μl, 1.5 μg/μl, 2 μg/μl in 10% ethanol and the control were used. To find the optimal concentration of 20E that could break pupal diapause of *B. minax*, the eclosion rates from these different concentrations were recorded.

### Pupal development after 20E-treatment by photomicrographs

The aim of this experiment was to monitor pupal development after 20E-treatment. The pupae samples used for this experiment were collected from the wild at the end of November, 2014, thus, they had already entered into pupal diapause. 20E was applied as reported above to break the pupal diapause. Following this treatment, pupae from all groups were dissected to record their developmental process. This was done by taking photomicrographs (Nikon DS-Fi1) every 3 days under a dissecting microscope (JSZ5BS). The pupal development was continuously monitored through this process until the adult emerged.

### Detection of the relative expression of 20E response genes by qRT-PCR

Early diapausing pupae, collected at the end of November, 2014, were ground with liquid nitrogen 1 h, 2 h, 4 h, 8 h, 12 h, 24 h, 48 h, 72 h and 96 h, after 20E and control treatments. Then RNA was extracted from the different samples with the use of the RNA extraction kit RNAiso Plus (TaKaRa). Reverse transcription of the total RNA into cDNA was carried out using RevertAid First Strand cDNA Synthesis Kit (Fermentas, EU) through reverse transcription PCR. These samples were either used immediately or stored at −20 °C. The specific primers for 20E response genes were designed for qRT-PCR using the Primer Premier 5 software (Table 2). These genes included *ecr*, *broad*, *foxo*, *bhr3*, *e74a* and *vrille* and α-TUB as a reference gene^24^,^41^. Then, relative expressions of some genes were detected after 20E treatment by qRT-PCR. Four replications were made.

### Statistical analysis

Data analysis was carried out using SPSS 16.0 (SPSS Inc., Chicago, Illinois, USA) and Origin 9.0 (Electronic Arts Inc., Redwood, California, USA) softwares. Eclosion rates and duration between treatments were compared using One-way ANOVA followed Duncan *post-hoc* test. Independent t-test was used to compare the qRT-PCR results. Nonlinear Curve Fit was done by using Origin 9.0 software^23^.

## Additional Information

**How to cite this article**: Chen, Z. *et al.* Pupal diapause termination in *Bactrocera minax*: an insight on 20-hydroxyecdysone induced phenotypic and genotypic expressions. *Sci. Rep.* **6**, 27440; doi: 10.1038/srep27440 (2016).

## Figures and Tables

**Table 1:** Means ± SD of time of 50% adult emergence (Et_50_) and starting day of emergence (SDE) of *Bactrocera minax* at different dates of collection and 20E-treatment.

Date of collection and treatment	Concentrations (μg/μl)	Et_50_(day)	SDE (day)
11/20/2014	CK	124.9 ± 12.24aA	103.4 ± 13.19aA
	1	44.2 ± 1.10b	41.6 ± 0.89b
	1.5	44.8 ± 2.78b	43.0 ± 3.81b
	2	43.4 ± 1.14b	40.4 ± 1.95b
12/19/2014	CK	95.1 ± 7.46aB	78.6 ± 8.27aB
	1	45.2 ± 0.45b	42.4 ± 0.89b
	1.5	45.2 ± 0.45b	41.2 ± 1.30b
	2	45.0 ± 0.71b	41.0 ± 0.71b
1/19/2015	CK	65.2 ± 1.10aC	52.8 ± 7.29aC
	1	44.4 ± 0.89b	40.2 ± 1.48b
	1.5	45.6 ± 1.52b	41.0 ± 0.71b
	2	44.8 ± 1.30b	40.4 ± 0.55b
2/24/2015	CK	48.6 ± 1.34aD	42.4 ± 1.52aD
	1	44.2 ± 1.10b	38.2 ± 1.79b
	1.5	44.2 ± 1.64b	38.6 ± 1.34b
	2	43.6 ± 1.82b	37.8 ± 0.84b
3/23/2015	CK	43.4 ± 2.30aD	39.6 ± 0.55aD
	1	41.2 ± 0.45b	39.4 ± 0.55a
	1.5	41.0 ± 0.71b	39.0 ± 0.00a
	2	41.0 ± 0.00b	39.2 ± 0.45a
4/20/2015	CK	32.0 ± 0.82E	25.0 ± 0.82E

The pupae were maintained at a constant temperature of 22 ± 1 °C. Different lowercase letter shows significant difference between 20E-treatment and control groups in the same date, different uppercase letter shows significant difference between control groups at different dates (*p* < 0.05, ANOVA).

**Table 2:** Primer sequences used for qRT-PCR.

Gene	Primer sequence (5′ → 3′)	Fragment length (bp)
bhr3	GCCATAACCGTAGGGAC AAGAAACAGCGTGAGAAA	192
e74a	CCCTGCCACCAACGACAA ACGCACCTGCTGCCCTAA	277
ecr	TACTTCCGATGAGCAGG GCGTATTATGAGCACCC	181
broad	CGTTCACCAGCCGAGTCAT CGCCACCGCTAAAGGAAGA	235
vrille	TACCGATGAAGAGGAAACG AACAGGTAACAGCGTCCAG	110
foxo	CTCGCTGTTCGGATAGG GGTCAAACTGCCAAAGG	253
α-TUB	CAATGGCTGTGGTGTT GTTGTGCCCAAGGATG	187
